# Supplementary material for: The genome sequence of the Nickerl’s Fritillary, Melitaea aurelia Nickerl, 1850 (Lepidoptera: Nymphalidae)
Source: Wellcome Open Res. 2025 Aug 22;10:459. [Version 1] doi: 10.12688/wellcomeopenres.24749.1 (PMC12495394; doi:10.12688/wellcomeopenres.24749.1)
Supplement: Peer review history

# Reviewer report: Reviewer response for version 1

**DOI:** 10.21956/wellcomeopenres.27266.r133558

**Published:** 2025-10-03

**Authors:** Sina Beier

**Affiliations:** 1 MRC Toxicology Unit, Cambridge, UK

The authors present a high quality genome of the Nickerl's Fritillary. It has been assembled to full chromosomal resolution using long read PacBio Sequencing paired with Hi-C sequencing for scaffolding and haplotyping. 

 As the specimen was female, only the W sex chromosome is missing from the data, with the Z chromosome and mitochondrium included.

 The data is finished to high quality and will provide a valuable resource for studies of this endangered but highly locally adapted species.

 It would be beneficial to have a supplementary map of the breaks and joins from manual curation to improve reproducibility.

Are sufficient details of methods and materials provided to allow replication by others?

Partly

Is the rationale for creating the dataset(s) clearly described?

Yes

Are the datasets clearly presented in a useable and accessible format?

Yes

Are the protocols appropriate and is the work technically sound?

Yes

Reviewer Expertise:

bioinformatics, genomics

I confirm that I have read this submission and believe that I have an appropriate level of expertise to confirm that it is of an acceptable scientific standard.

# Reviewer report: Reviewer response for version 1

**DOI:** 10.21956/wellcomeopenres.27266.r133556

**Published:** 2025-09-23

**Authors:** Laurence Despres

**Affiliations:** 1 Universite Savoie Mont Blanc, Chambéry, Auvergne-Rhône-Alpes, France; 2 Laboratoire d'Ecologie Alpine (Ringgold ID: 56837), Université Grenoble Alpes, Grenoble, Auvergne-Rhône-Alpes, France

The authors report on the chromosome-level, haplotype-resolved genome sequence from a female specimen of Nickerl’s Fritillary *Melitaea aurelia* (Nymphalidae). The final assembly includes 31 chromosomes : 30 autosomes and the chromosome Z. The W chromosome was not recovered and the female is supposed to be Z0. The mitogenome was also assembled. The quality of the assembly was further assessed by the proportion of complete BUSCO genes recovered, which is very high (~98% of Lepidoptera database BUSCO genes).

 The genome assembly is of good quality and was constructed using appropriate methods : Pac Bio HiFi long reads (35-fold coverage) and Hi-C data to confirm scaffolding, following the high standard of the Darwin Tree of Life Project for quality checking and results presentation.

 This high quality genome assembly will provide a reference genome for further genomic studies on this diversified genus of butterflies.

Are sufficient details of methods and materials provided to allow replication by others?

Yes

Is the rationale for creating the dataset(s) clearly described?

Yes

Are the datasets clearly presented in a useable and accessible format?

Yes

Are the protocols appropriate and is the work technically sound?

Yes

Reviewer Expertise:

evolutionary genomics

I confirm that I have read this submission and believe that I have an appropriate level of expertise to confirm that it is of an acceptable scientific standard.
